# Technology-Enhanced Classroom Activity Breaks Impacting Children’s Physical Activity and Fitness

**DOI:** 10.3390/jcm7070165

**Published:** 2018-06-29

**Authors:** Heidi Buchele Harris, Weiyun Chen

**Affiliations:** School of Kinesiology, University of Michigan, Ann Arbor, MI 48109, USA; bucheleh@yahoo.com

**Keywords:** real-time physical activity, wearable technology, fitness, Fitbits

## Abstract

Background: This study examined the effects of a 4-week technology-enhanced physical activity (PA) interventions on students’ real-time daily PA and aerobic fitness levels. Methods: 116 fifth-graders were assigned to one intervention group (*n* = 31) participating in daily physical activity engaging the brain with Fitbit Challenge (PAEB-C), another intervention group (*n* = 29) wearing Fitbits only (Fitbit-O) daily, five days per week, or the comparison group (*n* = 56). Four-week real-time PA data were collected from the intervention students via Fitbase. Three groups were pre- and post-tested aerobic fitness. Results: The PAEB-C students showed significantly higher steps and minutes of being very active and fairly active (*F* = 7.999, *p* = 0.014, *ŋ* = 0.121; *F* = 5.667, *p* = 0.021, *ŋ* = 0.089; *F* = 10.572, *p* = 0.002, *ŋ* = 0.154) and lower minutes of being sedentary daily (*F* = 4.639, *p* = 0.035, *ŋ* = 0.074) than the Fitbit-O group. Both Fitbit groups exhibited significantly greater increases in aerobic fitness scores than the comparison group over time (*F* = 21.946, *p* = 0.001, *ŋ* = 0.303). Boys were more physically active and fit than girls. Conclusions: Technology-enhanced PA intervention was effective for improving real-time PA and aerobic fitness.

## 1. Introduction

To obtain physical and mental health benefits, children are recommended to participate in 60 or more minutes of moderate-to-vigorous physical activity (MVPA) per day, and to demonstrate a healthy level of aerobic fitness [1,2,3]. However, one in three children did not meet the recommended daily MVPA minutes, and failed to meet aerobic fitness standards [4,5,6]. To address the critical concerns, health professionals have advocated that schools provide realistic settings for PA intervention [1,2,3,4,5,6,7]. More than 95% of youth are enrolled in schools and spend about half of their waking hours in schools [1,2,3,4,5,6]. Therefore, introducing physical activity (PA) to each school day may be beneficial in helping children to meet daily physical activity guidelines and improve aerobic fitness [1,2,3,4,5,6].

Integrating PA into daily classroom breaks is an important strategy for increasing daily PA levels of children during school [8,9,10,11,12,13]. A growing number of classroom-based PA intervention studies have shown that students who participated in daily 10-min classroom physical activity (i.e., TAKE 10!) or 15 min of physically active academic lessons (i.e., Physical Activity Across the Curriculum (PAAC)), on a regular basis, achieved significantly greater levels of daily MVPA compared to control students [10,11,12,13]. Further, Carlson et al. [12] found that ten-minute classroom PA breaks, in addition to physical education and recess, significantly increased the likelihood of obtaining 30 min of PA per day during school. Ma et al. [13] found that daily 4-min high intensity classroom PA breaks increased PA levels during school. However, most of studies did not examine the effects of the classroom PA breaks on improving children’s fitness levels [8,9,10,11,12,13]. Empirical studies show that physical fitness is one of the enabling factors that provide the physical foundations necessary for enjoyable and successful PA engagement in youth [14,15,16,17]. Physically fit children are willing to engage in PA and to maintain their PA behaviors, whereas physically unfit children tend to be physically inactive [14,15,16,17]. Importantly, these links are reciprocal, as children’s participation in PA provides opportunities for improving physical fitness as well [14,15,16,17]. 

Wearable technology has been used as a self-monitoring tool for promoting physical activity participation [18,19,20,21]. In the Futurestep study by Mikkola et al. [20], the results indicated that students’ use of Polar heart rate monitors increased an awareness of their own fitness level and physical activity level and improved their motivation for PA participation. In a systematic review of activity monitors and PA, Lubans et al. [21] found that twelve out of fourteen studies using activity monitors significantly increased PA levels in terms of steps, distances, durations, and intensities. Fitbit is specifically designed to work at the individual level by providing detailed personalized data, such as minute-by-minute data, and composite data on steps, distance traveled, MVPA, heart rate, and intensity [18,19,20]. Fitbit has been evidenced to be a reliable and valid self-monitor, self-evaluation, and motivational tool to facilitate an individual’s engagement in PA [18,19,20,21]. Lubans [21] notes that although technologies may not be a solution to our global health epidemic, they could be a way to facilitate behavioral change at an individual level. 

To the best of our knowledge, Fitbit has not been integrated into the classroom PA break intervention studies, nor has Fitbit been used as a daily intervention strategy to promote real-time PA levels and aerobic fitness levels in school-aged children. To fill the gaps, this study aimed to investigate the impact of the technology-enhanced classroom-based PA intervention on daily real-time PA levels and aerobic fitness levels. Our research hypotheses are (1) students in the two intervention groups: Physical Activity Engaging the Brain + Fitbit Challenge (PAEB-C) group, and Fitbit Only (Fitbit-O) group, will meet the recommended daily MVPA minutes; and (2) students in the two intervention groups will show a greater increase in aerobic fitness levels compared to the control group over time. The significance of this study lies in integration of Fitbits into daily classroom-based activity breaks and assessing students’ daily real-time PA levels over the course of the interventions. 

## 2. Methods

### 2.1. Participants and Research Design

Participants were fifth-grade students recruited from five classes in two elementary schools, matched by minority status and percentage of students receiving free and reduced lunch. A quasi-experimental design was used to assign one school to the experimental school and another school to the control school. In the experimental school, one fifth-grade class was assigned to the Physical Activities Engaging the Brain + Fitbit Challenge (PAEB-C) condition, and another was assigned to the Fitbit Only (Fitbit-O) condition. This study was conducted over the course of seven weeks. The first week was used for recruitment. The second (pre-test) and last weeks (post-test) were used to administer aerobic fitness tests to the students, and the four weeks in the middle were for the intervention.

Prior to the data collection, approvals from the Institutional Review Board and the school district were granted (HUM00102732). A copy of the study guidelines and a consent form were sent to the students’ parents/guardians. Then, a second letter was sent to home if no consent was returned by the end of the week. Parents’ signed consents were secured for the study prior to asking assent of the children. Children were given the written assent form just prior to the study. The classroom teacher read, out loud, the assent form, which described the purpose of the study and the student’s involvement in this study. A total of 96.9% of the students from the PAEB-C group (*N* = 31), 87.5% of the students from the Fitbit-O group (*N* = 29), and 87.7% in the control group (*N* = 56) consented to participate in this study. As a result, this included 116 fifth-grade students aged 10–11 years (57 girls vs. 59 boys). In the intervention school, 60% of the participants self-identified as a race other than white, with 30% African American. At the comparison school, 48% self-identified as a race other than white, with 19% African American. 

### 2.2. Treatment

#### 2.2.1. Fitbit-O Group

The Fitbit Charger + Heart Rate^TM^ tracker was used as a self-monitoring and self-motivating tool for students to participate in PA daily. The Fitbit Charger + Heart Rate^TM^ tracker is the device that uses a non-invasive wireless sensor on the wrist to measure heart rate. The Fitbit Charger + Heart Rate^TM^ device relies on an accelerometer to offer direct and immediate feedback in terms of steps, distance, floors climbed, and heart rate. Thus, the students in the Fitbit-O group wore their Fitbit Charger + Heart Rate^TM^ device daily, five school days per week for four weeks. They received immediate as well as weekly feedback, to monitor their own progress and the progress of their classmates. 

#### 2.2.2. PAEB-C Group 

The PAEB-C was designed to deliberately use both sides of the body, in unison and apart from each other, to coordinate both sides of the body and activate both hemispheres of brain. While the teacher showed a six-minute PAEB activity video once a day after the students had been sitting for 20 min, the students followed the video to immediately perform the PAEB activity for five days per week over the four-week intervention. The QuickTime videos were labeled Day 1 through Day 20. During the first week of the intervention, the PAEB were done very slowly, while in weeks 2 and 3, the activities were slightly faster. Finally, during the week 4, the speed was further increased. First, fine motor movements were rhythmically repeated eight, then four time, and last two, first in unison, and then opposite each other. Next, patterned hand movements focused on changing direction, going forward, sideways, up and down, also in the same rhythmic format. Then, gross motor movements included making figure eights, by simultaneously pairing arm movements in the same direction, by changing the direction and having the arms go in opposite directions. Lastly, gross motor skills utilized the entire body movement. For example, children went from a split to a squat stance, first in unison, then in opposite directions, so when the video instructor jumped sideways, the participants were encouraged to squat. These series of PAEB sequences were done throughout the intervention. The teacher reported missing the PAEB for 3 days (85% of the time).

In addition, the students wore their Fitbit Charger + Heart Rate^TM^ device daily, five school days per week for four weeks, following the same procedures used in the Fitbit-O group, except for use of Fitbit Challenge. Regarding the Fitbit Challenge, the PAEB-C students were encouraged to set their own individual goals each week. For example, during the first week of the intervention, the students were encouraged to increase their steps each day by 2000, with the goal of reaching at least 10,000 steps a day. Also, the PAEB-C students were informed of the daily and weekly challenges. The Fitbit Challenges came in the form of a log sheet, on which students were encouraged to record their PA to see if they meet their weekly challenges. The challenges included counting steps and setting a goal, estimating how many steps the whole classroom would take, setting a goal based on distance (miles), and a climbing challenge (floors). Each Monday, the classroom totals were sent to the teacher, highlighting the previous week’s goals. On each Monday, the classroom teachers were given reports that showed weekly averages for steps and distance traveled and floors climbed for their classes. 

#### 2.2.3. Control Group

The students in the control group neither wore the Fitbit and nor participated in any PA-related classroom breaks. Instead, they had regular classroom breaks based on the school schedule.

### 2.3. Data Collection

#### 2.3.1. Fitbits

Prior to giving the Fitbit Charger + Heart Rate^TM^ devices to the students, the investigator taught the students (1) where to put their Fitbits on the battery charger on Fridays; (2) how to check the battery; (3) if their Fitbit’s battery was low they need to charge it; and (4) how to immediately see their real-time steps, distance, calories burned, and the heart rate on the Fitbits they are wearing. Also, the investigator explained the wearing protocols: wear the Fitbit Charger Heart Rate^TM^ device from Monday through Friday at all times, but students were allowed to take the device off when bathing and during other water activities. In addition, they were told that they would not get in trouble if they took them off, but they were encouraged to wear them as often as possible. Students in the Fitbit-O group and in the PAEB-C group wore their Fitbits day and night from Monday morning (as they arrived at the classroom), until Friday afternoon, whereupon they put the Fitbits in the charging station before they left for home on the weekend during the four weeks of the intervention. 

On each Friday during the four weeks of the intervention, after students had left the school, the researcher went to the school and uploaded the information from the Fitbits onto a secure Fitbit Software database, Fitabase. This is a password protected site which is accessible only by the researcher and the data management team from Fitabase. The researcher recorded which Fitbits had been placed on the charging station, and which Fitbits were missing. The teachers were asked to remind their students to bring the Fitbits in on Fridays so that the data could be recorded. Teachers were also given printouts of the classroom’s average number of steps and minutes, as well as the classrooms total number of steps and distance traveled. Fitbits were collected after students had spent twenty days in the schools wearing them. 

#### 2.3.2. Fitness Assessment

Physical education teachers in both schools provided the study team with the results from an aerobic fitness assessment. The intervention school and the control school pre- and post-tested students’ aerobic fitness using the FitnessGram^®^ test (i.e., Progressive Aerobic Cardiovascular Endurance Run (PACER), one-mile run) during weeks 2 and 7 of the study. The FitnessGram test is a validated and reliable health-related fitness assessment toolkit designed by Cooper Institute [22]. The PACER and one-mile run were used to assess levels of cardiovascular endurance [22]. The PACER test and/or one-mile run test were used by PE teachers in each school for the fifth-grade students’ report cards. All of the students in both schools participated in the fitness assessments.

### 2.4. Data Analysis

This study used average daily steps of the 20 days collected from the Fitbit Charger + Heart Rate^TM^ trackers. Then, the data was translated into composite measures of sedentary, light active (LA), fairly active (FA), very active (VA) minutes, and steps via the Fitabase software. These were determined based upon metabolic equivalents (METs). METS are organized by the World Health Organization (WHO, 2015) into working versus resting metabolic rates. A unit of 1 MET is equivalent to sitting (sedentary), 2–3 METs are considered light activity, 4–6 METs is fairly active, and greater than 6 METs is considered very active. Daily data from the Fitbits was excluded from analysis if the student took fewer than 1000 steps or if they had fewer than 840 min of wearing the Fitbit. This resulted in losing an average of one day per person during the four-week study, or an average of 3.97 (±4.83) steps and 0.72 (±1.19) minutes for the Fitbit-O group and 1.87 (±2.21) steps and 0.90 (±1.56) minutes for the PAEB-C group. Neither group lost individual students (*N* = 60). Daytime sedentary minutes were calculated by subtracting the age-based average of 9 hours of sleep per night (WebMD, 2016). Therefore, 540 sedentary minutes were subtracted before sedentary averages were calculated. A ratio of fairly and very active minutes/total activity minutes was created. Descriptive statistics of each variable was computed for the two Fitbit groups. The Multivariate Analysis of Variance (MANOVA) was used to analyze if there is an overall significant difference in the five PA variables and sedentary variable between the two Fitbit groups controlling for gender and race. Subsequently, the Analysis of Variance (ANOVA) was conducted separately for the steps, very active (VA) minutes, fairly active (FA) minutes, and light active (LA) minutes, sedentary, and fairly and very active (FVA) minutes between the two groups. Additionally, an interaction effect was performed to see if gender and race moderated physical activity levels. Partial eta squared was calculated to determine the effect size of the intervention effect on each Fitbit variable. 

Students’ aerobic testing scores used to group into high fit (HF), healthy fitness zone (HFZ), and low fit (LF) based on the FitnessGram standards for healthy fitness zone for boys and girls [22]. Then, each fitness zone was coded as: 3 = HF, 2 = HFZ, and 1 = LF. Descriptive statistics of the coded fitness scores for the three groups were computed. A composite of HFZ and HF was used to determine the percentages of students who were at or above the healthy fitness zones (HFZs). The percentages of students meeting the HFZs by each group at the pre- and the post-test were calculated. A 2 (pre-test vs. post-test) × 3 (PAEB-C, Fitbit-O, and the control) ANOVA was conducted while controlling for gender and race. Subsequently, post hoc comparison method was analyzed to determine if there were any significant increases in HFZs between the two groups at a time. All statistical analyses were conducted with IBM SPSS statistics 24 and a significant level of *p* < 0.05 was set.

## 3. Results

### 3.1. Daily Real-Time Physical Activity Levels between the Two Intervention Groups

Table 1 presents the descriptive statistics of each Fitbit variable between the two groups. 

The PAEB-C group showed higher numbers of average daily real-time steps and higher average daily real-time minutes in very active (VA), fairly active (FA), and light active (LA) variables compared to the students in Fitbit-O group. Further, the PAEB-C group were fairly and very active (FVA) for about 31 min, while the Fitbit-O group were FVA for 15 min daily. By contrast, the PAEB-C group had lower average daily sedentary minutes compared to the Fitbit-O group. 

The results of the MANOVA revealed an overall significant difference in average daily steps, VA minutes, FA minutes, LA minutes, and sedentary minutes when controlling for gender and race between the two groups (*F* = 2.418, *p* = 0.039, *ŋ* = 0.215). Subsequently, the ANOVA revealed that the PAEB-C group took significant more daily steps than the Fitbit-O group (*F* = 7.999, *p* = 0.014, *ŋ* = 0.121). Likewise, the PAEB-C group spent significant more minutes being VA (*F* = 5.667, *p* = 0.021, *ŋ* = 0.089), FA (*F* = 10.572, *p* = 0.002, *ŋ* = 0.154), and FVA (*F* = 11.701, *p* = 0.001, *ŋ* = 0.168) daily than did the Fitbit-O group. No significant difference in LA minutes between the two groups was found (*F* = 0.707, *p* = 0.404, *ŋ* = 0.012). By contrast, the Fitbit-O group spent significant more minutes being sedentary per day than the PAEB-C group (*F* = 4.639, *p* = 0.035, *ŋ* = 0.074). Table 2 presents descriptive statistics of five Fitbit variables by groups and gender.

No significant interaction of group by gender in each PA variable and sedentary minutes was found. However, regardless of intervention type, boys were significantly more likely to participate in VA minutes (*F* = 6.383, *p* = 0.014, *ŋ* = 0.099), FA minutes (*F* = 7.408, *p* = 0.009, *ŋ* = 0.113), and FVA minutes (*F* = 7.167, *p* = 0.010, *ŋ* = 0.110). By contrast, no gender difference was found in light minutes (*F* = 0.172, *p* = 0.680, *ŋ* = 0.003) and sedentary minutes (*F* = 0.252, *p* = 0.617, *ŋ* = 0.003). 

### 3.2. Intervention Effects on Aerobic Fitness Levels

Table 3 shows the descriptive statistics of average aerobic fitness scores and percentages of meeting the HFZs among the three groups at the pre- and the post-test. 

At the pre-test, only 26% of the PAEB-C and Fitbit-O students were in HFZs, while 39% of the control students were in the HFZs. The two Fitbit groups’ mean aerobic fitness score was 1.26, while the control group was 1.52. By contrast, at the post-test, 61% of the PAEB-C students and 56% of the Fitbit-O students were in HFZs, while 26% the control students were in HFZs. The PAEB-C’s average aerobic fitness score was 1.61 and the Fitbit-O’s was 1.56, whereas the control group’s was 1.32. 

**Figure 1:**
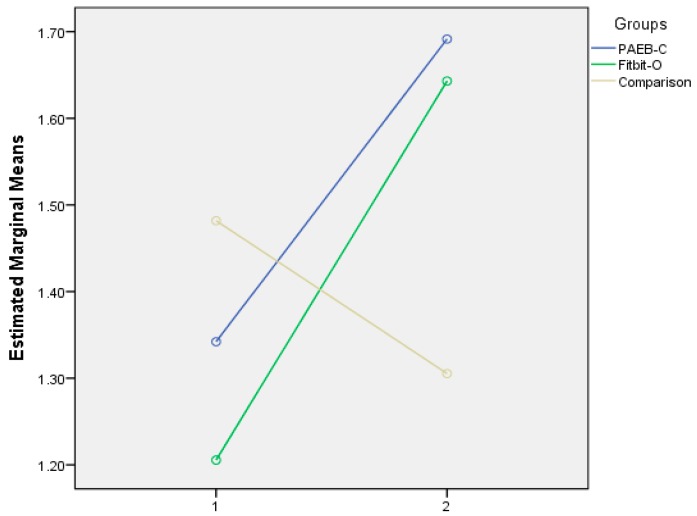
Mean scores of aerobic fitness among the three groups from pre- to post-test.

The results of the repeated measure ANOVA revealed significant main effect of time in aerobic fitness score (*F* = 19.273, *p* = 0.000, *ŋ* = 0.160), indicating the three groups showed significant improvement in the aerobic fitness testing score from pre- to post-test. Further, the repeated measure ANOVA revealed a significant interaction between time × treatment (*F* = 21.946, *p* = 0.000, *ŋ* = 0.303). Subsequently, the post hoc analysis revealed significant comparisons in the mean fitness scores from pre- to post-test between the PAEB-C and the control group (*F* = 29.327, *p* = 0.000), and between the Fitbit-O and the control group (*F* = 25.007, *p* = 0.000), but no significant difference between the two Fitbit groups (see Figure 1). 

## 4. Discussion

This study was central to examining the effects of the technology-enhanced physical activity interventions on real-time daily PA and aerobic fitness levels in school-aged children. The students in the PAEB-C group, who engaged in daily classroom-based activity breaks in conjunction with the Fitbit Challenge program, exceeded 10,000 steps daily, on average, and took, on average, 2206 more steps/day than the Fitbit-O group. Consistent with the results, a systematic review by Dobbins, Husson, DeCorby, and LaRocca [23] concluded that school-based physical activity interventions were more likely to improve MVPA minutes by as little as five and up to forty-five minutes. Similarly, classroom-based activity break studies have shown improved PA levels [10,11,12,13,24]. 

Another promising result was that the PAEB-C group had 107 fewer sedentary minutes/day than the Fitbit-O group. Supporting the previous studies, the results indicated that engaging the students in daily classroom-based activity breaks may be a feasible way to reduce their sedentary time during school day [10,11,12,13]. Reducing prolonged sedentary behaviors have a significant impact on a child’s overall health, as well as cognitive functions (i.e., attention, concentration, and information processing) [10,11,12,13]. However, the PAEB activities alone could not account for these differences. Since the PAEB activities are mostly fine and small gross motor skills, they predominantly fell into the light and fairly active categories. This suggests that the Fitbit Challenge may have played an important role in increasing steps and reducing sedentary minutes. Children engaging in both PAEB activities and the Fitbit Challenges were motivated by them to be more active than the Fitbit-O group.

It is important to note that the PAEB-C group, on average, spent 30.94 min, and the Fitbit-C group spent 15.47 min engaging in very active (vigorous) and fairly active (moderate) PA daily over the course of four weeks. Their daily real-time physical activity minutes are far lower than the recommended daily 60 min or more of MVPA by the 2008 Physical Activity Guidelines [4]. However, the PAEB-C group engaged 248 min, and the Fitbit-O group engaged 231 min, in being light active, daily. This is consistent with the findings by Van der Niet et al. [24] who found that students’ time was spent mostly in PA that consisted of light PA. 

In addition, this study showed the gender discrepancy in daily MVPA. Though the PAEB-C group took more very active and fairly active minutes than the Fitbit-O group, most of these differences can be attributed to time spent in very active and fairly active PA by boys. Boys were more likely to participate in very active and fairly active PA than girls throughout the study. In a study of 1111 fourth- and fifth-grade students’ daily PA in year 1, and 1012 fourth- and fifth-grade students’ daily PA in year 2 of the Healthy Kids and Smart Kids project, Chen et al. [14] found that boys self-reported they were more physically active than girls in daily PA during school and outside of school in both years. Similarly, Ridgers, Salmon, Parrish, Stanley, and Okely [25] found the boys were more likely to participate in a higher amount of MVPA minutes than girls. 

As expected, the two Fitbits groups showed greater increases in aerobic fitness compared to the control group from pre- to the post-test. After the four-week interventions, 61% of the PAEB-C and 56% of the Fitbit-O students were in the HFZs. Similarly, Chen et al. [14] reported 59% of 265 fifth-grade students met the HFZ standards in PACER test at the end of participating in one-school year Healthy Kids and Smart Kids project. By contrast, the percentages of meeting the HFZs dropped from 39% to 26% for the comparison school. These changes occurred during a time when seasonal changes sometimes act to reduce fitness levels [9]. Fedewa et al. [9] found that though students participate in daily classroom-based PA breaks, their daily average steps decreased during winter season. However, this study showed that the seasonal factor seemed not to negatively influence the two Fitbits groups’ aerobic fitness scores at the post-test. Rather, the two Fitbit groups showed significant increases in aerobic fitness scores. The results suggest that the students’ wearing the Fitbit daily, checking their real-time daily steps taken, and self-monitoring their progress toward meeting the weekly-based goal, are effective intervention strategies for improving their aerobic fitness levels, in addition to merely engaging in classroom activity breaks. The results might be attributed to the reciprocal relationship between regular PA participation and physical fitness [15]. Supporting this point, Chen et al. [14] found that children’s total weekly PA minutes in and outside school were significantly associated with healthy level of aerobic fitness. Similarly, previous studies found a significant association between amount of PA and aerobic fitness in school-aged children [14,15,16,17]. Another possible reason for the two Fitbits groups’ significant increases in aerobic fitness would be that Fitbits do allow for students to immediately monitor their real-time heart rates. The study suggests that Fitbits can be used to encourage students to spend a greater amount of time in their target heart rate zones. In addition, the overall excitement of wearing a Fitbit for four weeks, the wearable device effect, might also play a role in increasing students’ aerobic fitness levels. 

The limitation of the study was that fitness testing data were based upon one-mile run and the PACER tests. Originally, both schools planned to do the one-mile test, but the Fitbit school underwent construction during the study, which did not allow them to take the one-mile run at the post-test. However, both tests were typically used to assess aerobic fitness levels based on the gender- and age-specific standards for low fit, healthy fitness zone, and high fit in the FitnessGram^®^ [22]. Another limitation of this study was not using the Fitbit Charger + Heart Rate^TM^ device to collect the students’ average daily heart rate data. The future study could use this essential data to examine the participants’ daily heart rates patterns over the course of the intervention, and how their average daily heart rates are associated with increased physical activity participation and aerobic fitness levels. Also, this study was not focused on examining how the students’ wearing the Fitbit device daily motivated their participation in physical activities and improved their aerobic fitness. A future study may examine how wearing the Fitbits daily will influence participants’ intrinsic and extrinsic motivations for physical activity, which in turn, is conducive to developing habitual physical activity behaviors. 

## 5. Conclusions

The students’ daily real-time MVPA minutes did not meet the recommended 60 min of MVPA daily. However, the integration of Fitbits into daily classroom activity breaks were effective intervention strategies for engaging students in more minutes of MVPA and reducing their sedentary minutes compared to the intervention strategy that merely integrated Fitbits into their daily life. Boys were more likely to be physically active than girls, when wearing the Fitbits. Use of Fitbits as a daily and real-time intervention strategy along with daily classroom-based activity breaks for four weeks significantly increased proportions of students meeting HFZs. 

## Figures and Tables

**Table 1:** Mean steps and minutes of physical activity between the two intervention groups. Fitbit-O, Fitbit Only; PAEB-C, Physical Activity Engaging the Brain + Fitbit Challenge.

	Fitbit-O	PAEB-C
	*M ± SD*	*M ± SD*
Steps	8954.54 ± 2518.59	11151.78 ± 3421.68
Very Active (VA)	4.09 ± 5.00	7.96 ± 7.32
Fairly Active (FA)	11.28 ± 8.00	22.98 ± 17.75
Light Active (LA)	230.57 ± 82.23	247.78 ± 76.33
Sedentary	454.34 ± 203.23	347.75± 179.51
Fairly and Very Active (FVA)/Total Activity	1.3 ± 1	2.9 ± 2.3

**Table 2:** Descriptive statistics of five Fitbit variables by groups and gender.

	Fitbit-O	PAEB-C
	*M ± SD*	*M ± SD*
Steps		
Boys	9139.64 ± 1872.23	12,221.84 ± 4161.87
Girls	8814.95 ± 2839.23	10,270.55 ± 2460.06
Very Active (VA)		
Boys	5.47 ± 5.77	10.83 ± 7.05
Girls	3.36 ± 4.54	5.61 ± 6.84
Fairly Active (FA)		
Boys	14.35 ± 10.16	29.97 ± 17.22
Girls	9.67 ± 6.33	17.22 + 10.90
Light Active (LA)		
Boys	249.41 ± 49.17	223.42 ± 73.07
Girls	220.66 ± 94.92	267.84± 75.10
Sedentary		
Boys	380.64 ± 167.19	385.78 ± 195.47
Girls	491.43 ± 214.01	318.30 ± 164.90

**Table 3:** Average aerobic fitness scores and percentage of healthy fitness zones (HFZs) at the pre- and post-test by groups.

	Pre-Test		Post-Test	
	*M ± SD*	*% HFZs*	*M ± SD*	*% HFZs*
PAEB-C	1.26 ± 0.45	26%	1.61 ± 0.50	61%
Fitbit-O	1.26 ± 0.45	26%	1.56 ± 0.51	56%
Control	1.52 ± 0.71	39%	1.38 ± 0.71	26%
